# Hyaluronic Acid Nanoparticles for Immunogenic Chemotherapy of Leukemia and T-Cell Lymphoma

**DOI:** 10.3390/pharmaceutics14020466

**Published:** 2022-02-21

**Authors:** Vinu Krishnan, Vimisha Dharamdasani, Shirin Bakre, Ved Dhole, Debra Wu, Bogdan Budnik, Samir Mitragotri

**Affiliations:** 1 School of Engineering and Applied Sciences, Harvard University, Cambridge, MA 02138, USA; vinu@udel.edu (V.K.); vimishadharamdasani@gmail.com (V.D.); shirin.bakre@gmail.com (S.B.); ved.dhole@gmail.com (V.D.); debrahwu@gmail.com (D.W.); 2 Wyss Institute of Biologically Inspired Engineering, Harvard University, Boston, MA 02115, USA; 3 Mass Spectrometry Proteomics and Research Laboratory, FAS Division of Science, Harvard University, Cambridge, MA 02138, USA; bbudnik@mcb.harvard.edu

**Keywords:** hyaluronic acid nanoparticles, leukemia, lymphoma, drug delivery

## Abstract

Ratiometric delivery of combination chemotherapy can achieve therapeutic efficacy based on synergistic interactions between drugs. It is critical to design such combinations with drugs that complement each other and reduce cancer growth through multiple mechanisms. Using hyaluronic acid (HA) as a carrier, two chemotherapeutic agents—doxorubicin (DOX) and camptothecin (CPT)—were incorporated and tested for their synergistic potency against a broad panel of blood-cancer cell lines. The pair also demonstrated the ability to achieve immunogenic cell death by increasing the surface exposure levels of Calreticulin, thereby highlighting its ability to induce apoptosis via an alternate pathway. Global proteomic profiling of cancer cells treated with HA–DOX–CPT identified pathways that could potentially predict patient sensitivity to HA–DOX–CPT. This lays the foundation for further exploration of integrating drug delivery and proteomics in personalized immunogenic chemotherapy.

## 1. Introduction

Combination chemotherapy has played an active role in treating blood cancers such as acute myeloid leukemia (AML) and T-cell lymphoma (TCL). Over the past couple of decades, significant effort has gone into understanding the biological basis of blood cancers. This accelerated the development and use of drug cocktails in clinical oncology [1,2,3,4,5,6,7,8,9]. The general guidelines for using such cocktails largely remain focused on combining (a) drugs with different mechanisms of action to counter multidrug resistance and (b) drugs with non-overlapping toxicities for administration at near-maximal dose [10,11,12,13]. Combinations have also been tested on a metronomic basis, although still at the maximum tolerated dose (MTD) for each drug. The rationale for using each drug at its MTD stems from the sigmoidal dose–response curves obtained in vitro where a narrow therapeutic window often suggests that “more is better” [14]. However, considering the way drugs interact within the body or reach the target site, administering cocktails based on MTD is not an optimal approach. While synergistic ratios are potentially beneficial, ratios that end up being antagonistic at the target site could harm the patient [15,16,17]. To achieve target cell accumulation of synergistic drug combinations, it is vital to maintain control over the differential pharmacokinetics, distribution, and metabolism of each drug following its systemic delivery in vivo. Nanotechnology has revolutionized disease diagnosis and therapy. In addition to applications in multimodal in vivo imaging for disease diagnosis or single-drug delivery, nanotechnology can be used to achieve the ratiometric delivery of multiple chemotherapeutic agents [18,19,20]. Previously, we have shown the ability to achieve this ratiometric control by conjugating drug pairs at synergistic ratios to the glycopolymer, namely Hyaluronic Acid (HA), to form HA nanoparticles [21,22,23]. HA can bind specifically to CD44, a cell surface receptor that is typically expressed at high levels on blood-cancer cells [24,25]. This could promote an increased uptake for the particles [21,23,26]. By incorporating the drug pair on to HA, the differential blood circulation properties, tissue biodistribution levels, and cell uptake rate of each drug are now normalized to the carrier itself. The advent of combinatorial cancer nanomedicine has therefore resulted in a variety of different nanoparticles including liposomes, polymers, polymer–drug conjugates, and dendrimers being tested for similar applications in combination drug delivery [9,27,28,29,30,31]. Such nanoparticulate carriers not only offer the benefit of combining poorly soluble drugs such as Camptothecin with their synergistic water-soluble partners but also ensures the pairs’ ratiometric stability during circulation and at the target site. Achieving precise control over drug loading within the same nanoparticle-based carrier during formulation and maintaining temporal drug release in vivo will offer significant therapeutic implications in clinical oncology.

Although combination chemotherapy can reduce the primary tumor burden, presence of dormant or residual cancer cells can result in an aggressive disease relapse. This underscores the importance of achieving cancer-specific immunity in patients for minimizing chances of a relapse. However, the immunosuppressive nature of chemotherapy makes it a challenge to achieve antitumor immunity, especially when combined with immunotherapies. Conventional chemotherapy was always known to induce tumor cell death via non-immunogenic mechanisms such as apoptosis [32]. However, studies have shown that chemotherapeutic drugs such as anthracyclines (doxorubicin), cyclophosphamide, mitoxantrone, oxaliplatin, and bortezomib, among others, have the ability to elicit an antitumor immunity response and thereby induce an immunogenic cell death [33,34,35,36,37]. The drugs can activate the innate immune response and elicit a tumor-specific adaptive immune response that damage the tumors. Thus, engineering drug combinations at ratios capable of inducing immunogenic cell death at significantly low doses could possibly impart a strong and sustained anti-tumor activity without being detrimental to the patient.

With the advent of personalized chemotherapy, global proteomic profiling is increasingly being carried out in patient-derived cell lines and tissues to provide insights into cancer classification, treatment regimens, and analyses of drug resistance [38,39,40]. However, to date, there have been very few studies that explore the effect of ratiometric drug delivery on the proteomic profile of blood cancers. Distinct ratios give rise to contrasting biological effects that are likely to manifest differently at the proteome level. Hence, an analysis of proteomic modulation is vital in understanding how to design effective drug combinations in the clinic.

Here, we propose an effective and personalized immunogenic chemotherapy by conjugating doxorubicin (DOX) and camptothecin (CPT) at different ratios to the HA polymer backbone (HA–DOX–CPT). Previously, HA nanoparticles have demonstrated efficacy in treating solid tumors in vivo [21,22,23]. DOX, an anthracycline, is widely used in the clinic to treat a variety of cancers. CPT, a highly potent anticancer drug, is hydrophobic and unstable in plasma, thereby limiting its use in the clinic. Furthermore, water-soluble analogs of CPT are known to have limited efficacy and can cause severe side-effects in patients [41]. DOX and CPT belong to the class of topoisomerase enzyme inhibitors II and I. Topoisomerase enzymes play a critical role during DNA transcription and cell replication [42,43]. Besides, the high expression levels of TOP 1 enzymes in cancer cells enable drugs such as CPT to achieve high-target specificity [44,45,46]. Consequently, the DOX–CPT combination has been widely explored as a combination chemotherapy in the clinic on account of its synergistic anticancer activity [47,48,49,50,51,52,53]. In this study, we incorporated the pair at different ratios onto HA and used its anticancer synergy to examine HA–DOX–CPT’s potential as an immunogenic chemotherapy.

We first establish a broad spectrum of the biological response for multiple cell lines, namely leukemia and T-cell lymphoma, which are both drug-sensitive and resistant types. For this, we perform a systematic evaluation of HA–DOX–CPT at different ratios over a range of concentrations. The most synergistic ratios are then assessed for their immunogenic activity in different cell lines. We then identified the key signaling pathways, which, when activated, can overcome drug resistance and induce immunogenic cell death with enhanced therapeutic efficacy. For this, we executed a small-scale proteomic workflow to obtain the quantitative proteomics profile of a multidrug-resistant blood-cancer cell line: HL-60/MX2 treated with HA–DOX–CPT. We identified up/downregulated proteins externalized by HL-60/MX2 based on the combined exploitation of mass spectrometry and bioinformatics tools. Eventually, this enabled us to identify biological pathways that could potentially predict patient sensitivity to HA–DOX–CPT-based personalized immunogenic chemotherapy.

## 2. Materials

Camptothecin (CPT) and 4-(dimethylamino)pyridine (DMAP) were purchased from Sigma-Aldrich (St. Louis, MO, USA). N-(3-Dimethylaminopropyl)-N′-ethylcarbodiimide hydrochloride (EDC) was purchased from Life Technologies, Carlsbad, CA, USA. Doxorubicin hydrochloride (DOX–HCl) was obtained from LC Laboratories (Woburn, MA, USA). Hyaluronic acid (HA) of 50 kDa MW was purchased from Creative PEGWorks (Winston Salem, NC, USA). Cell Titer Blue was purchased from Life Technologies, USA. All cell lines—leukemia and T-cell lymphoma (SI, Table S2)—were generously provided by Dr. David Weinstock, Department of Medical Oncology, Dana-Farber Cancer Institute and Harvard Medical School. The cells were cultured in Roswell Park Memorial Institute (RPMI) 1640 Medium (Life Technologies) supplemented with 20% fetal bovine serum (FBS) and maintained at 37 °C under a humidified atmosphere of 95% air and 5% CO_2_. Antibodies used for flow-cytometry analysis were obtained from Biolegend. Sephadex G-25 PD-10 columns were obtained from GE Healthcare Life Sciences (Piscataway, NJ, USA) and dialysis cassettes with 3500 MWCO were obtained from Life Technologies, USA. All other chemicals used for this study were obtained from Fisher Scientific and were of the highest possible grade commercially available.

## 3. Methods

### 3.1. Synthesis of HA–DOX–CPT

Single and dual-drug conjugates were synthesized via nucleophilic acyl substitution reactions (Figure S1). In total, 100 mg of 50 kDa MW HA was dissolved in a 2 mL 1:1 mixture of DI water and dimethyl sulfoxide at 40 °C, followed by the addition of DMAP/EDC at the 1:1 molar ratio relative to the monomer mass. After 30 min of activation, the drugs DOX and CPT were added at 0.4:1 and 0.2:1 molar ratios, respectively. For synthesizing HA–DOX–CPT at different ratios, DOX and CPT were added at varying amounts to the polymer as summarized in SI, Table S1. The products were then passed through a size exclusion chromatography column (Sephadex G-25 PD-10 desalting columns (5000 M.W. exclusion limit)) and left for overnight dialysis (3500 MWCO) against DI water. The lyophilized product was stored at 4 °C until further use and was reconstituted in PBS before use. The amounts of drugs incorporated were measured using fluorescence spectra at Ex/Em 470/590 for DOX and 370/448 nm for CPT.

### 3.2. Physical Characterization

#### 3.2.1. Size

The NanoSight LM10 system (NanoSight, Amesbury, UK) was used to assess the size of HA–DOX–CPT. For size analysis, the Nanosight system was supplemented with a fast video capture and Nanoparticle Tracking Analysis (NTA 2.3) software. The samples were measured by capturing videos set at a recording time of 30 s each with adjustments made to the manual shutter and gain at room temperature. Image processing was done using the NTA 2.3 software to record the size. For each sample, the instrument was recalibrated and the measurements were made in triplicates.

#### 3.2.2. In Vitro Release Studies

Lyophilized HA–DOX–CPT was reconstituted in PBS buffer (pH 7.4) at 1 mg/mL and incubated in Slide-A-Lyzer MINI dialysis devices (10,000 MWCO) at 37 °C for up to 5 days. The dialysis devices were inserted into microcentrifuge tubes with 1 mL PBS. In total, 100 µL of the release medium in microcentrifuge tubes was collected at the indicated time points and drug concentration was measured via fluorescence using the TECAN plate reader. Points for the cumulative release were obtained by dividing the amount of drug released each day with the mass for the initial input. All measurements were carried out in triplicates and the results are indicated as the mean ± SD.

### 3.3. In Vitro Toxicity Analysis

All 7 T-cell lymphoma and 7 leukemia cell lines were incubated with HA–DOX, HA–CPT, and HA–DOX–CPT (R0.8, R1.5, R12, and R25) over a range of serially diluted concentrations for 48 h and 72 h, respectively. For the dual-drug conjugates, the treatments were set up with respect to DOX concentrations. This was followed throughout the study. The cell lines were seeded in a 96-well cell culture plate at 50,000 cells per well and maintained in RPMI media (20% FBS) at 37 °C in a 5% CO_2_ atmosphere. The Cell Titer-Blue^®^ Viability Assay was used to measure the cell viability and was expressed as the percentage of viable cells relative to the survival of untreated cells (defined as the maximum cell viability). The combination index (CI) was then estimated from the dose–response curves plotted for the single and dual-drug conjugates. A value of CI less than 1 indicates synergism; CI = 1 indicates the additive effect; and CI > 1 indicates antagonism. The further a CI value is from 1, the more pronounced the drug interaction, i.e., synergism or antagonism.

### 3.4. In Vitro Anticancer Immunogenic and Drug-Resistant Marker Assay

Leukemia cell lines (HL-60, HL-60/MX2, MOLM-13, and THP-1) and T-cell lymphoma cell lines (Ki-JK, MTA:NK-LL, and FEPD) were seeded at a density of 50,000 cells/100 µL in RPMI media (20% FBS) and maintained at 37 °C in a 5% CO_2_ atmosphere. The cells were then incubated overnight with HA–DOX–CPT (5 µM DOX) and stained with anti-human Alexa Fluor^®^ 647 Calreticulin, Alexa Fluor^®^ 488 anti-Hsp70 or its isotype controls (Alexa Fluor^®^ 647 Mouse IgG2a and Alexa Fluor^®^ 488 Mouse IgG2a) prior to analysis via flow cytometry.

### 3.5. Protein Extraction from Cells

Dried samples were resolubilized in Covaris (Woburn, MA, USA) DF buffer and break with focused ultrasound by the Covaris S220 instrument with a 180 s cycle and 10% peak-to-peak power. After the cell break down procedure, all proteins were precipitated from the extraction solution by the methanol/chloroform cold precipitation procedure and all buffers were removed. Protein pellets were resolubilized in TEAB (triethyl ammonia bicarbonate) buffer, pH 7.5, for a further FASP based on 10 kDa filters (Pall, TX, USA) with reduction and alkylation steps using a previously established filter-assisted digestion protocol [54]. Digested proteins were labeled with TMT11plex (Tandem Mass Tags) labels (Thermo-Fisher, Dreieich, Hessen, Germany) according to the manufacturer’s protocol. After the labeling procedure, all samples were quenched with 5% hydroxyl solution and pooled together as one sample.

### 3.6. Hi pH Separation and Mass Spectrometry Analysis

Post digestion of TMT, the labelled set the samples were submitted for hiPH separation based on the Pierce™ High pH Reversed-Phase Peptide Fractionation Kit (Thermo-Fisher Scientific, Waltham, MA, USA). The peptides were divided into 40 fractions and dried in SpeedVac (Eppendorf, Hamburg, Germany). The fractions were then resuspended in 0.1% formic acid solution before injection to the mass spectrometer. Tribrid Lumos Orbitrap (Thermo-Fisher Scientific, Waltham, MA, USA) equipped with the UltiMate 3000 HPLC Nano tandem pump (Thermo-Fisher Scientific, Waltham, MA, USA as used for a single LC-MS/MS experiment. Using a 150 µm inner diameter microcapillary trapping column, the peptides were separated. The column was packed first with approximately 3 cm of C18 Reprosil resin (5 µm, 100 Å, Dr. Maisch GmbH, Ammerbuchm, Germany), followed by 50 cm of the PharmaFluidics analytical column (PharmaFluidics, Ghent, Belgium). A gradient from 5 to 27% ACN in 0.1% formic acid over 90 min at 200 nl/min was applied to achieve separation. A home-made electrode junction at the end of a microcapillary column and sprayed from stainless steel 4 cm needles (Thermo-Fisher Scientific, Waltham, MA, USA) was used to enable electrospray ionization by applying a voltage of 2 kV.

The Lumos Orbitrap instrument was operated in data-dependent mode for the mass spectrometry methods. The mass spectrometry survey scan was performed in the Orbitrap in the range of 410–1800 m/z at a resolution of 6 × 104, followed by the selection of the twenty most intense ions (TOP20) for HCD-MS2 fragmentation in the Lumos Orbitrap using a precursor isolation width window of 0.8 Th, the AGC setting of 50,000, and a maximum ion accumulation of 200 ms. Singly charged ion species were not subjected to HCD fragmentation. Collision energy was normalized and set to 37 V, and an activation of the time of 1 ms was set. Ions in a 10 ppm m/z window around ions selected for MS2 were excluded from further selection for fragmentation for 60 s and subsequently each precursor ion was selected for the CID type of fragmentation in ion trap part of the instrument with both 25 V energy and a max of 50 ms ion accumulation time.

Proteome Discoverer 2.4. (Thermo-Fisher Scientific, Waltham, MA, USA) software was used to analyze raw data. The Sequest HT algorithm was used for assignment of MS/MS spectra by searching the data against a protein sequence database. The Bacillus Subtilis database and other known contaminants such as human keratins and common lab contaminants were also searched. Sequest HT searches were performed using a 10 ppm precursor ion tolerance and required each peptides’ N-/C termini to adhere with Trypsin protease specificity, while allowing for up to two missed cleavages. Furthermore, 6-plex TMT tags on peptide N termini and lysine residues (+229.162932 Da) were set as static modifications while methionine oxidation (+15.99492 Da) was set as a variable modification. A target-decoy database search was applied to MS2 spectra with a false discovery rate (FDR) of 1% on the protein level. Filtering was performed using a percolator (64-bit version) [55]. For quantification, a 0.02 m/z window centered on the theoretical m/z value of each of the six reporter ions and the intensity of the signal closest to the theoretical m/z value were recorded. Reporter ion intensities were exported in the result file of the Proteome Discoverer 2.4 search engine as an excel table. The total signal intensity across all peptides quantified was summed for each TMT channel and all intensity values were adjusted to account for potentially uneven TMT labeling and/or sample handling variance for each labeled channel.

### 3.7. Statistical Analysis

All experiments were carried out in triplicates and results are indicated as the mean ± SD unless otherwise indicated. All graphs have been generated and analyzed using Prism non-linear regression software (GraphPad Software, San Diego, CA, USA). A *p* value of < 0.05 was considered significant.

## 4. Results

### 4.1. Physical Characterization of HA–DOX–CPT

**Figure 1:**
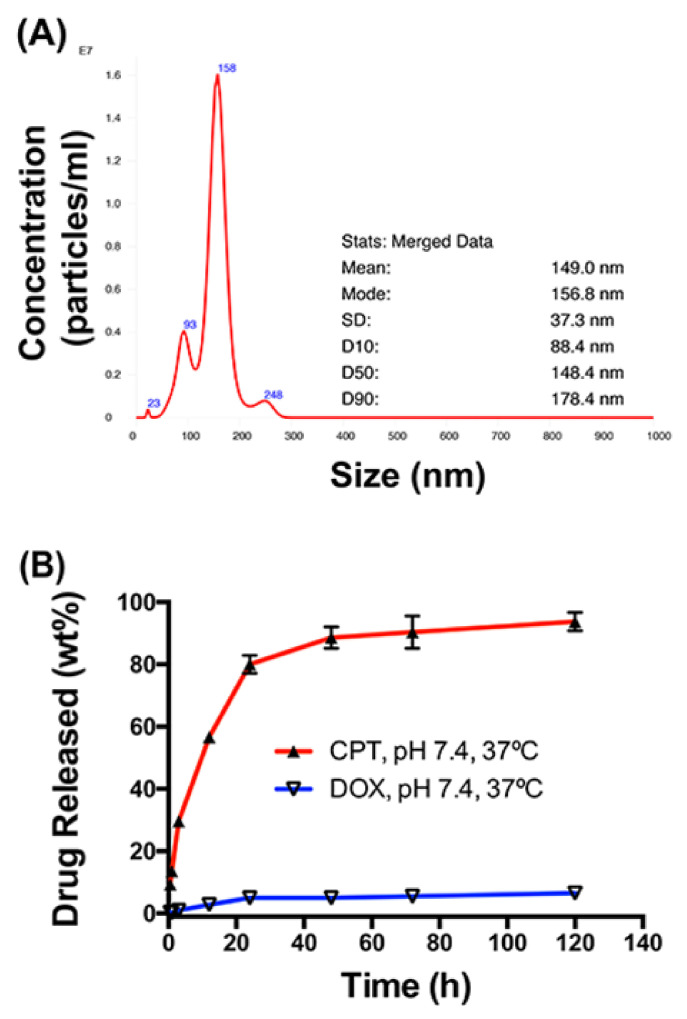
**Physical characterization of HA–DOX–CPT.** (**A**) NTA analysis revealed the mean average size to be at 149 nm with a fairly polydisperse size distribution. (**B**) In vitro release rates for HA–DOX–CPT in PBS at 37 °C. Each value represents the mean ± SEM (*n* = 3).

Chemotherapeutic drugs, namely doxorubicin (DOX) and camptothecin (CPT), were incorporated onto the HA polymer backbone at various molar ratios (R0.8, R1.5, R12, and R21, R = molar ratio of DOX:CPT). Nanoparticle Tracking Analysis (NTS) revealed the mean average size to be at 149 nm with a fairly polydisperse size distribution (Figure 1A). In vitro release rates of HA–DOX–CPT in physiological buffer conditions indicated that approximately 17.24 ± 0.92 wt% DOX and 94.15 ± 5 wt% CPT were released (Figure 1B). The differential release of both drugs from HA is attributed to the formation of an amide bond between the HA backbone and DOX, and an ester bond between HA and CPT [21].

### 4.2. Increased Synergistic Potency of HA–DOX–CPT

**Figure 2:**
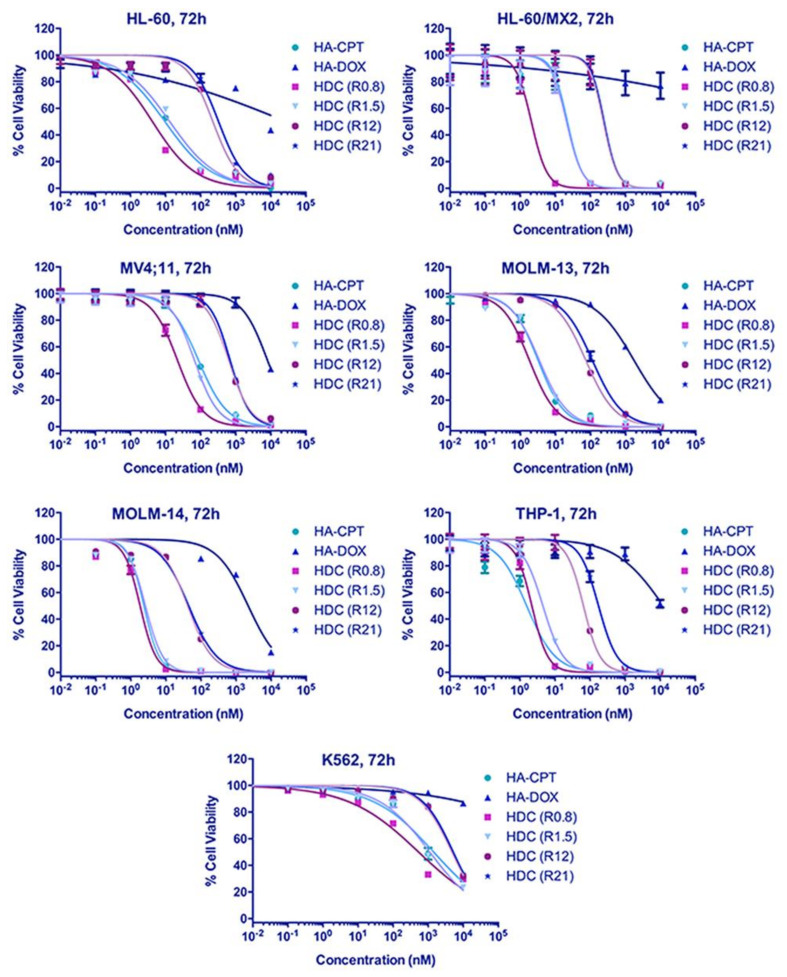
**Increased potency of HA–DOX–CPT against leukemia.** When treated with leukemia cells, the IC90 value of DOX in HA–DOX–CPT was reduced by at least 20-folds lower compared to HA–DOX and for CPT, it was at least two-folds lower than HA–CPT alone.

**Figure 3:**
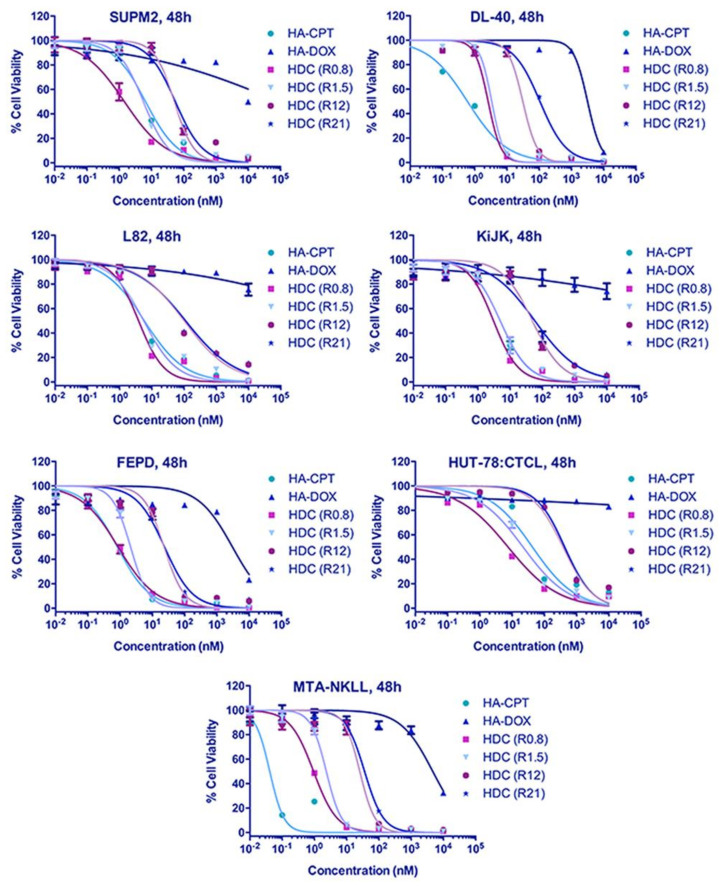
**Increased potency of HA–DOX–CPT against T-cell lymphoma.** When treated with T-cell lymphoma cell lines, the IC90 value of DOX in HA–DOX–CPT was reduced by at least 11-folds lower compared to HA–DOX alone and for CPT, it was two-folds lower than HA–CPT alone.

**Figure 4:**
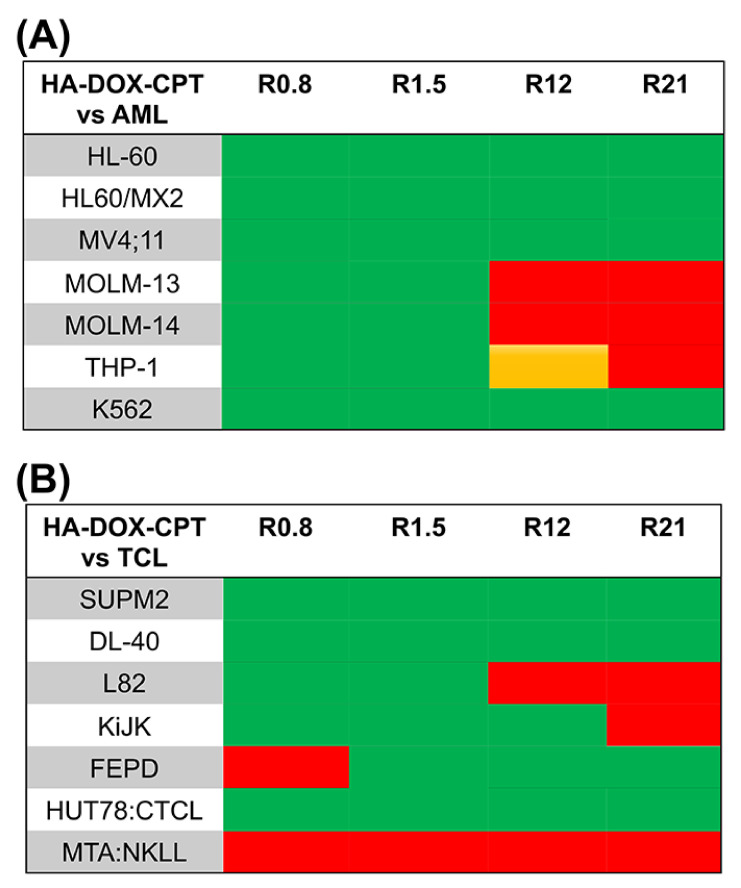
**Increased synergistic potency against leukemia and lymphoma with less toxicity towards normal lymphoblasts.** (**A**,**B**) The bioresponse heatmap impart information regarding the biological response of leukemia and T-cell lymphoma on account of DOX and CPT interactions when incorporated to HA. The map was generated off the CI values computed from the IC90 values obtained for single and dual-drug conjugates treated with leukemia and lymphoma cell lines. Green indicates a CI < 1 depicting synergism; orange for CI = 1 depicts an additive effect; and red indicates a CI > 1 depicting antagonism. HA–DOX–CPT induced a synergistic toxic effect on a majority of leukemia and lymphoma cell lines.

HA–DOX–CPT was highly effective against both leukemia and T-cell lymphoma cell lines. For the leukemia cell lines, IC90 values for DOX in HA–DOX–CPT were at least 20-folds lower compared to HA–DOX alone and for CPT, the values were at least two-folds lower than HA–CPT alone (Figure 2 and SI, Tables S4–S10). For the T-cell lymphoma cell lines, the IC90 values for DOX in HA–DOX–CPT were at least 11-folds lower compared to HA–DOX alone and for CPT, the values were at least two-folds lower than HA–CPT alone (Figure 3 and SI, Tables S11–S17). Furthermore, combination indices (CI) were estimated from the IC50 values of single and dual-drug treatments and used to generate a bioresponse heatmap (Figure 4A,B). A value of CI < 1 indicates synergism (green); CI = 1 suggests an additive effect (orange); and CI > 1 antagonism (red). The bioresponse heatmap depicted a highly synergistic interaction between DOX and CPT for a majority of leukemia and T-cell lymphoma cell lines, with the exception of FEPD and MTA-NKLL (Figure 4). On the account of its high efficacy and safety, HA–DOX–CPT R0.8 was chosen as the lead candidate and evaluated extensively in subsequent studies.

### 4.3. Anticancer Immunogenicity of HA–DOX–CPT

**Figure 5:**
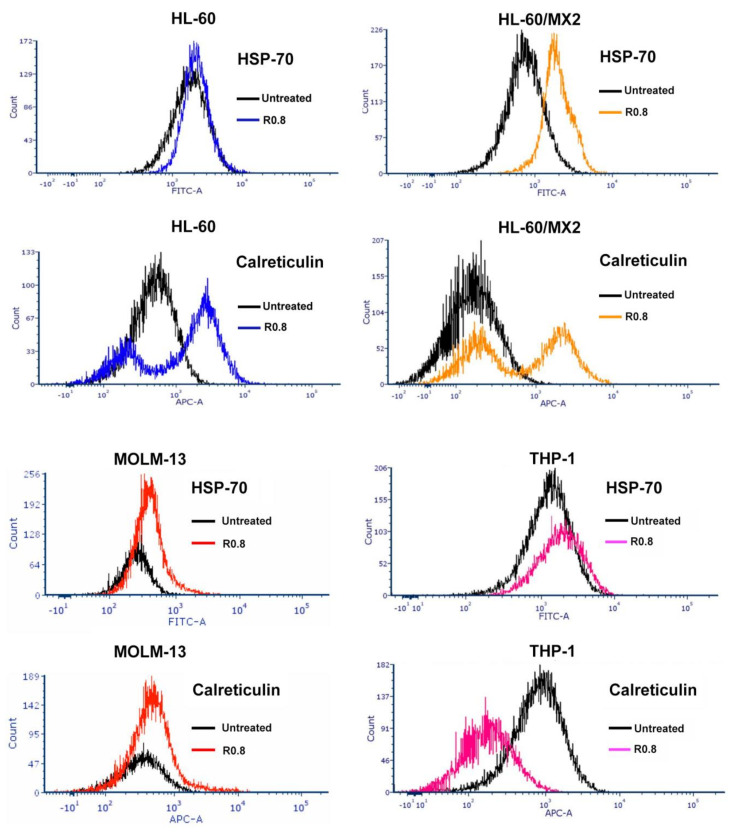
**HA–DOX–CPT induces anticancer immunogenic response in leukemia.** When treated with HA–DOX–CPT, surface exposure of Calreticulin (CRT) was upregulated for HL-60 and its multidrug-resistant variant HL-60/MX2. However, the levels remained at baseline for MOLM-13 and were reduced for THP-1, indicating varying degrees of immunogenic response to chemotherapy.

**Figure 6:**
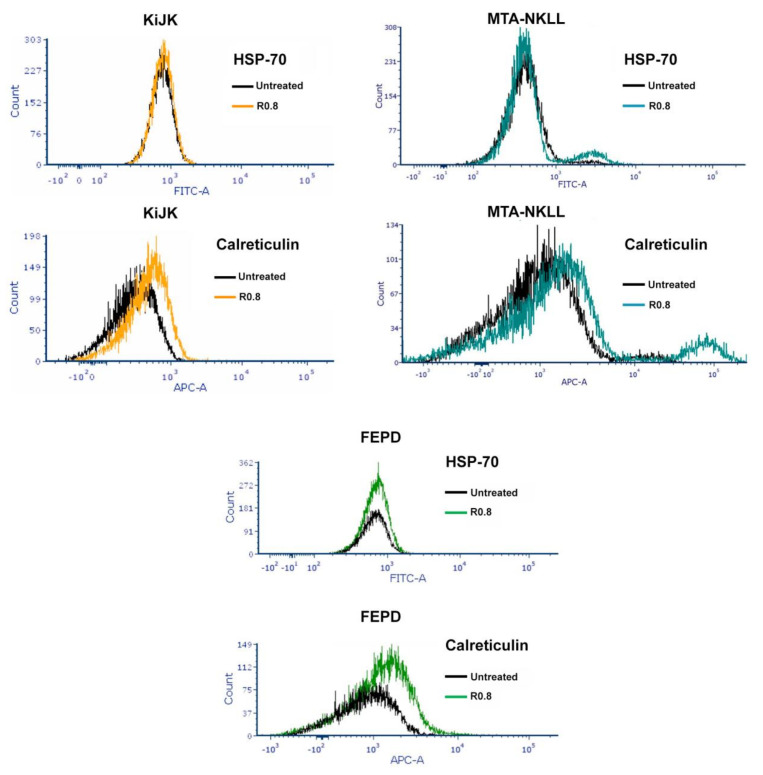
**HA–DOX–CPT induces anticancer immunogenic response in T-cell lymphoma cell lines.** When treated with HA–DOX–CPT, surface exposure of Calreticulin (CRT) was upregulated for the T-cell lymphoma cell lines tested while no change was observed for the stress-induced drug-resistant marker HSP-70.

HA–DOX–CPT upregulated the surface exposure of Calreticulin (CRT), an inducer of immunogenic cell death in HL-60 and its multidrug-resistant variant HL-60/MX2. However, the levels remained at the baseline for MOLM-13 and were reduced for THP-1 (Figure 5 and Table 1). For the T-cell lymphoma cell lines, the immunogenic response was actively upregulated (Figure 6 and Table 2). Interestingly, the stress-induced drug-resistant marker HSP-70 was upregulated for all the leukemia cell lines and none of the TCL cell lines tested.

### 4.4. Proteomic Profiling of DOX-Resistant Leukemia Cell Line (HL-60/MX2)

**Figure 7:**
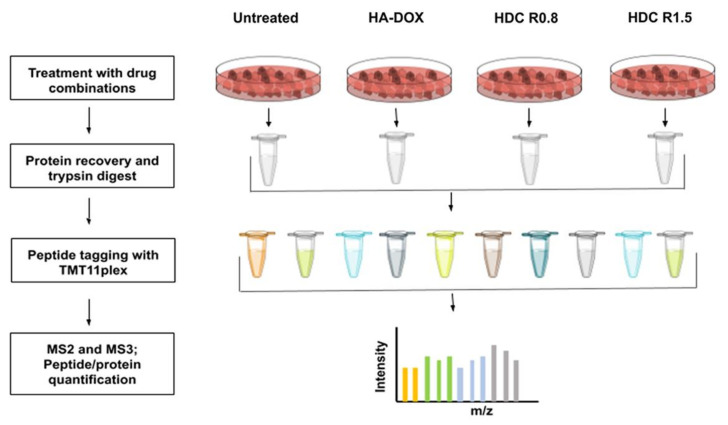
**Workflow for the proteomics analysis**. Biological replicates of the proteomes of HL-60/MX2 cells under different conditions were quantitatively mapped using TMT11plex reagents and Lumos Orbitrap instrument.

MS-based quantitative proteomic profiling was performed to compare the proteome expression levels of DOX-resistant HL-60/MX2 cells in four different conditions (outlined in Figure 7). The cells were treated overnight with HA–DOX, HA–DOX–CPT R0.8, and HA–DOX–CPT R15 at a concentration of 10 µM. Following the treatment, protein extraction, and tryptic digest, the samples were labelled with tandem mass tags (TMT). TMT labeling allows for the multiplexed relative quantification of proteins in different samples using high resolution MS. A total of 4000 proteins were quantified in the cell line and subsequent analyses were performed on this subset. Validation of hits was performed by analyzing the mRNA expression under the same experimental conditions via RT-qPCR (SI, Figure S3). The primer sequences are as given in SI, Table S20.

**Figure 8:**
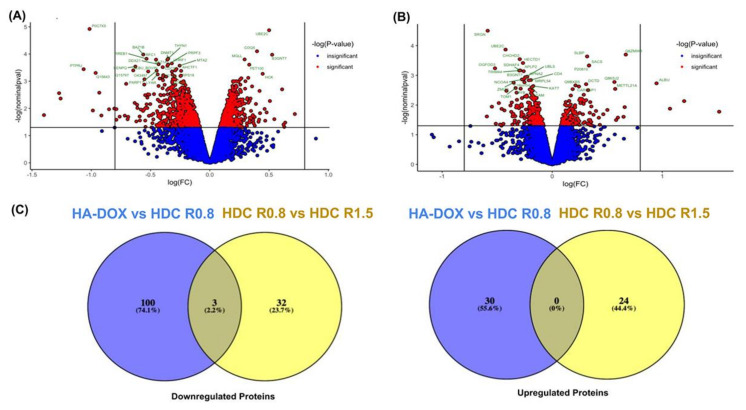
**Summary of the differentially expressed proteins after the indicated treatments.** (**A**,**B**) Volcano plot illustrates significantly differentially abundant proteins. The −log10 (Benjamini–Hochberg corrected *p* value) is plotted against the log2 (fold change). The non-axial vertical lines denote ±0.5-fold change while the non-axial horizontal line denotes *p* = 0.05, which is our significance threshold. (**A**) Differentially expressed proteins for HA–DOX vs. HA–DOX–CPT R0.8 at *p* value ≦ 0.05 (**B**) Differentially expressed proteins for HA–DOX–CPT R0.8 vs. HA–DOX–CPT R1.5 at *p* value ≦ 0.05. (**C**) Venn diagram illustrates the relative comparison of downregulated and upregulated proteins for HA–DOX vs. HA–DOX–CPT R0.8 and HA–DOX–CPT R0.8 vs. HA–DOX–CPT R1.

To graphically represent the t-test data, a volcano plot-log10 (*p* value) vs. log2 (fold change of HA–DOX/HA–DOX–CPT R0.8) or log2 (fold change of HA–DOX–CPT R0.8/HA–DOX–CPT R15) was constructed to graphically display the quantitative data (SI, Figure S4). Points above the non-axial horizontal line represent proteins with significantly different abundances (*p* < 0.05). Points to the left of the left-most non-axial vertical line denote protein fold changes of HA–DOX/HA–DOX–CPT R0.8 (Figure 8A) or protein fold changes of HA–DOX–CPT R0.8/HA–DOX–CPT R15 (Figure 8B) of less than −0.5. Similarly, points to the right of the right-most non-axial vertical line denote protein fold changes of HA–DOX/HA–DOX–CPT R0.8 (Figure 8A) or protein fold changes of HA–DOX–CPT R0.8/HA–DOX–CPT R15 (Figure 8B) of greater than 0.5. Change in the protein expression for the treatment group was compared to the untreated samples (control; SI, Figure S3). There were 20 proteins whose levels changed significantly in HA–DOX vs. HA–DOX–CPT R0.8 treatments and 28 proteins that changed while comparing HA–DOX–CPT R0.8 vs. R1.5 treatments. The Venn diagram (Figure 8C) shows the percentage of differentially expressed proteins that are unique to the treatments.

### 4.5. Bioinformatic Analysis—Identification and Enrichment of Differentially Expressed Proteins

**Figure 9:**
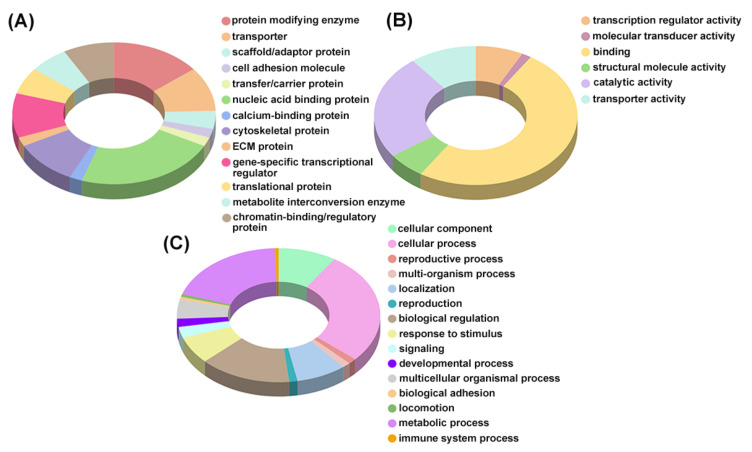
**GO analysis of all the identified proteins by Proteome Discoverer software.** (**A**) Protein class analyses of the identified proteins. (**B**) Molecular function analyses of the identified proteins. (**C**) Biological processes analyses of the identified proteins.

**Figure 10:**
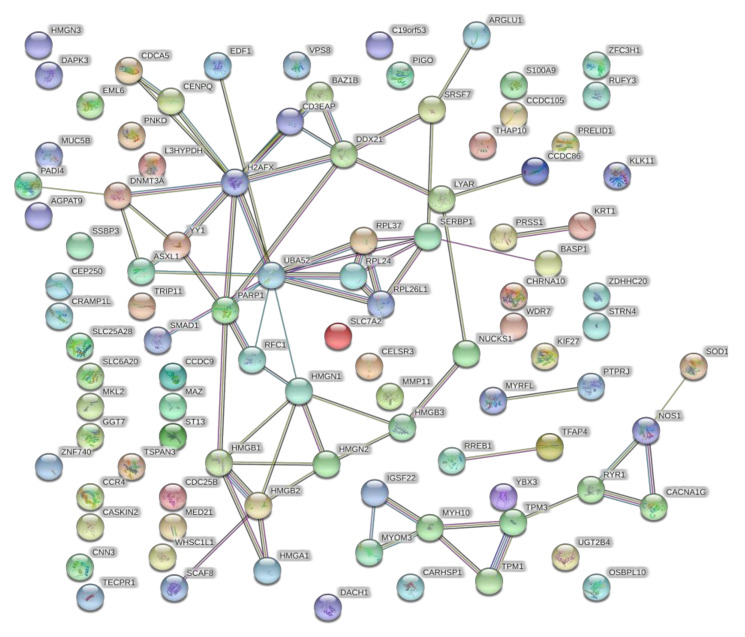
**Biological interaction network/STRING analysis** screenshots from the STRING website, showing results obtained upon entering proteins that are upregulated in HA–DOX–CPT R0.8 when compared to HA–DOX-treated cells.

Differentially abundant proteins were subjected to the GO classification via the Panther Classification System database to identify associated biological processes, molecular functions, and the protein class. The proteins enriched in HA–DOX–CPT R0.8-treated cells belonged to the nucleic acid binding, gene-specific transcriptional regulator, and chromatin binding regulatory protein class. The molecular functions included binding, transporter activity, and transcription regulatory activity. GO analysis (Figure 9) and STRING analysis (Figure 10) identified proteins associated with chromosomal regulation and reorganization as the most significantly enriched cellular processes during treatment with HA–DOX–CPT R0.8 and relative to HA–DOX.

## 5. Discussion

From a clinical perspective, this study demonstrates the ability to use highly potent, synergistic, and immunogenic drug combinations to overcome multidrug resistance in leukemia and T-cell lymphomas. By virtue of proteomic analysis, we quantified the differentially expressed proteins and identified key signaling pathways that are activated in response to the ratiometric delivery of DOX and CPT. The study identifies biomarkers that could possibly predict the clinical response to HA–DOX–CPT, thereby setting a path for its development in personalized chemo-immunogenic therapy.

A polydisperse size distribution was revealed for HA–DOX–CPT in PBS with a mean size ranging from 149 nm (Figure 1A). With TEM and AFM, a polydisperse spherical morphology was observed for these nanoparticles in PBS [23]. When incorporated on HA, the IC90 values of the single-drug nanoparticles increased for DOX and CPT compared to the values obtained for the free drugs (SI, Figure S2, and SI, Table S3A). Such differences in IC90 values could possibly arise from (a) differences in uptake mechanisms for the free vs. nanoparticle form of the drugs and (b) lag in drug availability due to different release rates from the carrier. The particles are likely to be taken up via endocytosis, while free drugs diffuse freely through the cell membrane. The differences in drug availability are further supported by comparing the release rates for both DOX and CPT from HA–DOX–CPT (Figure 1B). However, the difference did not affect the synergistic potency of HA–DOX–CPT for most of the leukemia and T-cell lymphoma cell lines (Figure 2, Figure 3 and Figure 4A,B, and SI, Tables S4–S17). A synergistic interaction was observed between DOX and CPT for cell lines irrespective of its inherent drug-resistant properties.

Increased surface exposure of Calreticulin (CTR) in response to a drug is an important damage-associated molecular pattern (DAMP) for anticancer immunogenicity [56,57]. DOX is known to activate and increase the early surface exposure of Calreticulin (CTR), an endoplasmic reticulum chaperone protein in cancer cells [33,58,59,60,61]. This triggers an “eat-me” signal from the tumor, resulting in its phagocytosis by the dendritic cells (DC) accompanied by further recruitment and maturation of macrophages, thereby activating signaling pathways responsible for the downstream activation of antitumor immunity [59,60]. Treatment with HA–DOX–CPT increased the CTR surface exposure in most cell lines tested (Figure 5 and Figure 6). This suggests that HA–DOX–CPT can achieve tumor cell death via DOX and CPT-mediated inhibition of topoisomerase activity and the induction of antitumor immunogenicity. DOX is also known to upregulate the stress inducible heat shock protein 70 (HSP70) in cancer cells. Increased upregulation of HSP70 confers drug resistance in stress-induced cancer cells and enhances their ability to regrow aggressively [62,63,64]. Treatment with HA–DOX–CPT increased HSP70 levels in leukemia cells and not in T-Cell lymphoma (Figure 5 and Figure 6). However, HA–DOX–CPT demonstrated an ability to overcome the obstacle of HSP70-induced drug resistance via a synergistic interaction between DOX and CPT, highlighting the importance of ratiometric drug delivery in stress-induced cancers.

Recent advances in proteomics provide a powerful approach to study the global cellular effects of a drug or drug cocktail. We quantified more than 4000 cellular proteins and identified a total of 48 proteins whose levels changed in response to HA–DOX and HA–DOX–CPT treatment with the multidrug resistant cell line HL-60/MX2. Principal component analysis revealed strong patterns within the dataset SI, Figure S5. Drug resistance is a complex process that is potentially the result of multiple and overlapping mechanisms that are also affected by other biological factors. This determines the degree of response to therapy and supports the rationale for personalized chemotherapy, which requires cocktails that take into consideration the individual variability and are then optimized for a given patient and type of cancer.

GO analysis pertaining to the molecular function revealed binding, catalytic activity, a transporter, and a transcription regulator activity as affected in response to treatment with HA–DOX–CPT (Figure 9B and Figure 10). GO analysis for the biological processes revealed that cells’ metabolic and immune system processes were affected, among many others (Figure 9C and Figure 10). The major protein classes that were affected belonged to nucleic acid binding and cell adhesion molecules (Figure 9A and Figure 10). An apparent difference in the protein expression was observed for HL-60/MX2 cells treated with HA–DOX–CPT R0.8 and R1.5. One such interesting protein was the ubiquitin-conjugating enzyme 2C (UBE2C). The protein was upregulated (SI, Table S19) when cells were treated with HA–DOX–CPT R1.5 and downregulated (SI, Table S18) when treated with HA–DOX–CPT R0.8. UBE2C is a member of the E2 ubiquitin-conjugating enzyme family and plays a significant role in the ubiquitin conjugation. It is involved in mitotic cyclin B degradation, leading the cell cycle to undergo transition from the M phase to the G1 phase. It is likely that the aberrant UBE2C overexpression may affect ubiquitin homeostasis, giving rise to uncontrolled cell proliferation. Based on the literature, patients with higher UBE2C levels show a reduced overall survival that correlates with poor clinical outcomes [65]. It was interesting to note that a change in the molar ratio of the drug combination from 0.8 to 1.5 causes overexpression of a potential cancer biomarker such as UBE2C.

One of the hallmarks of cancer progression is chromosomal instability (CIN), a source of genetic variation in altered chromosomal structures or its number. Chromosomal reorganization was depicted as a key pathway during the GO analysis of treatment groups HA–DOX vs. HA–DOX–CPT. When compared with HA–DOX, chromatin assembly and disassembly was upregulated in the cells treated with HA–DOX–CPT. Many chemotherapeutic agents are known to upregulate CIN, which leads to the apoptosis of aberrant cells. However, contradictory findings while studying the effect of CIN on therapeutic response and drug resistance have also been reported [66]. Some studies associate high CIN with a better therapeutic response and others associate it with therapeutic resistance. Hence, careful patient selection will be crucial to determine the effect of HA–DOX–CPT-based combination therapy.

## 6. Conclusions

Despite major advances in proteomics, the use of this technique in analyzing the efficacy of ratiometric drug delivery is still in its infancy. This is an excellent approach for the discovery of predictors not just for individual drug treatments but also for personalized combination therapies. Our proteomic studies in blood-cancer cells have revealed multiple candidates that might serve as biomarkers of prognosis and drug resistance. In the future, these findings can be utilized as a resource to guide the design of combination drug delivery while identifying mechanisms or pathways that promote multi-drug resistance and promote enhanced therapeutic efficacy. Future studies will be focused on validating the chemo-immunogenic effect of HA–DOX–CPT in drug-resistant tumor models in vivo. However, challenges exist in ensuring that the chemo-immunogenic effect of this combination at specific ratios is retained at the target site. Since DOX and CPT have relatively different pharmacokinetic properties, normalizing their circulation times to that of the nanoparticle carrier would be a first step. Incorporating the drug pair onto the carrier would involve a systematic approach to formulation design and optimization. In addition to ensuring the stability of these hyaluronic acid nanoparticles during circulation, achieving control over its drug release for the desired pharmacodynamic effects following its cell uptake would be vital. By integrating “proteomics” with “designer combinatorial NPs”, traditional cancer nanomedicine that focuses on targeting and disease management could undergo a paradigm shift for a whole-body level of effective immunoprotection against cancer relapse.

## Figures and Tables

**Table 1:** **HA–DOX–CPT may have the potential to overcome HSP-70-induced drug resistance in AML cells.** When treated with HA–DOX–CPT, an increased upregulation of HSP70 was observed for most of the cell lines tested. Upregulation of HSP70 can confer drug resistance in stress-induced cancer cells, enhancing their ability to proliferate rapidly. However, as indicated by the CI values, HA–DOX–CPT did overcome the obstacle of HSP70-induced drug resistance via a synergistic interaction between DOX and CPT, highlighting the importance of ratiometric drug delivery in stress-induced cancers.

HA–DOX–CPT R0.8, 48 h	HSP-70 Expression	Calreticulin Expression	˜CI
**HL-60**	increase	increase	**0.3 (synergistic)**
**HL-60/MX2**	increase	increase	**0.09 (synergistic)**
**MOLM-13**	no change	no change	**0.5 (synergistic)**
**THP-1**	increase	decrease	**0.1 (synergistic)**

**Table 2:** **HA–DOX–CPT upregulates Calreticulin but has no effect on HSP-70 in T-cell lymphoma cells.** When treated with HA–DOX–CPT, no upregulation of the stress-induced drug-resistant marker HSP-70 was observed, but the immunogenic response monitored by Calreticulin expression levels was actively upregulated in T-cell lymphoma.

HA–DOX–CPT R0.8, 48 h	HSP-70 Expression	Calreticulin Expression	˜CI
**KiJK**	no change	increase	**0.4 (synergistic)**
**MTA-NKLL**	no change	increase	**>>>1 (highly antagonistic)**
**FEPD**	no change	increase	**1.2 (antagonistic)**

## Data Availability

Not applicable.

## Supplementary Materials

The following supporting information can be downloaded at: https://www.mdpi.com/article/10.3390/pharmaceutics14020466/s1, Figure S1: Reaction scheme and conditions for HA polymer-drug conjugates at different ratios; Figure S2: Dose-response curves and IC50 values for free DOX and free CPT treatment with HL-60; Figure S3: QPCR Validation. The mRNA expression of two representative proteins (SRSF3 and CD63) from the proteomics list was validated; Figure S4: Principal Component Analysis was performed to bring out strong patterns within the dataset; Figure S5: Differentially expressed proteins in treated vs untreated samples. Volcano plot illustrates significantly differentially abundant proteins. The −log10 (Benjamini–Hochberg corrected *P* value) is plotted against the log2 (fold change). (A) Untreated vs HA-DOX (B) Untreated vs HA-DOX-CPT R0.8 (**C**) Untreated vs HA-DOX-CPT R1.5; Table S1: The drugs were incorporated onto HA in a series of molar ratios by varying the amount of drugs added and reaction time; Table S2: List of cell lines used; Table S3: Dose-response curves and IC50 values for free DOX and free CPT treatment with HL-60; Table S4: Summary of IC90 and C.I. values for HA-DOX-CPT when treated with Acute Myeloid Leukemia cells (HL-60). HA-DOX-CPT achieves a high synergy with C.I values <1; Table S5: Summary of IC90 and C.I. values for HA-DOX-CPT when treated with drug-resistant Acute Myeloid Leukemia cells (HL-60/MX2). DOCTOR achieves a very high synergy with C.I values <<1; Table S6: Summary of IC50 and C.I. values for DOCTOR when treated with Acute Monocytic Leukemia Cells (MV4; 11). HA-DOX-CPT achieves high synergy with C.I values <1; Table S7: Summary of IC90 and C.I. values for DOCTOR when treated with Acute Myeloid Leukemia Cells (MOLM-13). HA-DOX-CPT achieves high synergy for ratios R0.8 and R1.5 with C.I values <1; Table S8: Summary of IC90 and C.I. values for DOCTOR when treated with Acute Myeloid Leukemia Cells (MOLM-14). HA-DOX-CPT achieves high synergy for ratios R0.8 and R1.5 with C.I values <1; Table S9: Summary of IC90 and C.I. values for DOCTOR when treated with Acute Monocytic Leukemia Cells (THP-1). HA-DOX-CPT achieves high synergy for ratios R0.8 and R1.5 with C.I values <1; Table S10: Summary of IC90 and C.I. values for DOCTOR when treated with BCR-ABL1+ Chronic Myeloid Leukemia Cells (K562). HA-DOX-CPT achieves very high synergy with C.I values <<1; Table S11: Summary of IC90 and C.I. values for DOCTOR when treated with Alk+ anaplastic large cell lymphoma (SUP-M2). HA-DOX-CPT achieves high synergy with C.I values <1; Table S12: Summary of IC90 and C.I. values for DOCTOR when treated with Alk- anaplastic large cell lymphoma (DL-40). HA-DOX-CPT achieves high synergy with C.I values <1; Table S13: Summary of IC90 and C.I. values for DOCTOR when treated with Alk+ anaplastic large cell lymphoma (L82). HA-DOX-CPT achieves high synergy for ratios R0.8 and R1.5 with C.I values <1; Table S14: Summary of IC90 and C.I. values for DOCTOR when treated with Alk+ anaplastic large cell lymphoma (Ki-JK). HA-DOX-CPT achieves high synergy for ratios R0.8, R1.5 and R12 with C.I values <1; Table S15: Summary of IC90 and C.I. values for DOCTOR when treated with Alk- anaplastic large cell lymphoma (FEPD). HA-DOX-CPT achieves high synergy for ratios R1.5, R12 and R21 with C.I values <1; Table S16: Summary of IC90 and C.I. values for DOCTOR when treated with T-cell lymphoma (HUT-78:CTCL). HA-DOX-CPT achieves very high synergy for all ratios with C.I values <<1; Table S17: Summary of IC90 and C.I. values for DOCTOR when treated with NK/T-cell lymphoma (MTA:NK-LL). HA-DOX-CPT achieves very high synergy for all ratios with C.I values <<1; Table S18: Select protein expression level comparison between HA-DOX and HA-DOX-CPT treated HL-60/MX2 identified by MS. Proteins upregulated in HA-DOX treatment, downregulated in HA-DOX-CPT R0.8 treatment (Orange). Proteins downregulated in HA-DOX treatment, upregulated in HA-DOX-CPT R0.8 treatment (Blue); Table S19: Select protein expression level comparison between HA-DOX-CPT R0.8 and HA-DOX-CPT R1.5 treated HL-60/MX2 identified by MS. Proteins upregulated in HA-DOX-CPT R0.8 treatment, downregulated in HA-DOX-CPT R1.5 treatment (Yellow). Proteins downregulated in HA-DOX-CPT R0.8 treatment, upregulated in HA-DOX-CPT R1.5 treatment (Green); Table S20: Primer sequences for qPCR validation.
