# Supplementary material for: Hyaluronic Acid Nanoparticles for Immunogenic Chemotherapy of Leukemia and T-Cell Lymphoma
Source: Pharmaceutics. 2022 Feb 21;14(2):466. doi: 10.3390/pharmaceutics14020466 (PMC8874923; doi:10.3390/pharmaceutics14020466)
Supplement: Supplementary file 1 [file pharmaceutics-14-00466-s001.zip › pharmaceutics-1539645-supplementary.pdf]

pharmaceutics

MDPI

Supplementary Materials: Hyaluronic Acid Nanoparticles for Immunogenic Chemotherapy of Leukemia and T-Cell Lymphoma

Vinu Krishnan, Vimisha Dharamdasani, Shirin Bakre, Ved Dhole, Debra Wu, Bogdan Budnik and Samir Mitragotri

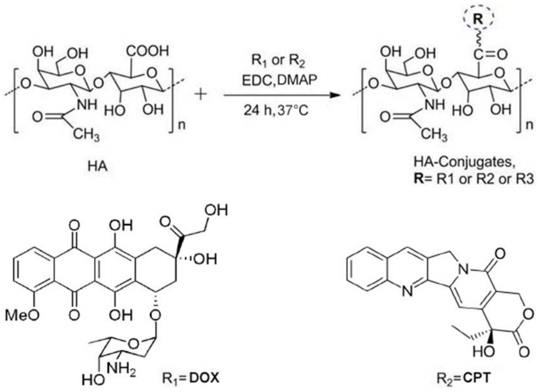

Figure S1. Reaction scheme and conditions for HA polymer-drug conjugates at different ratios.

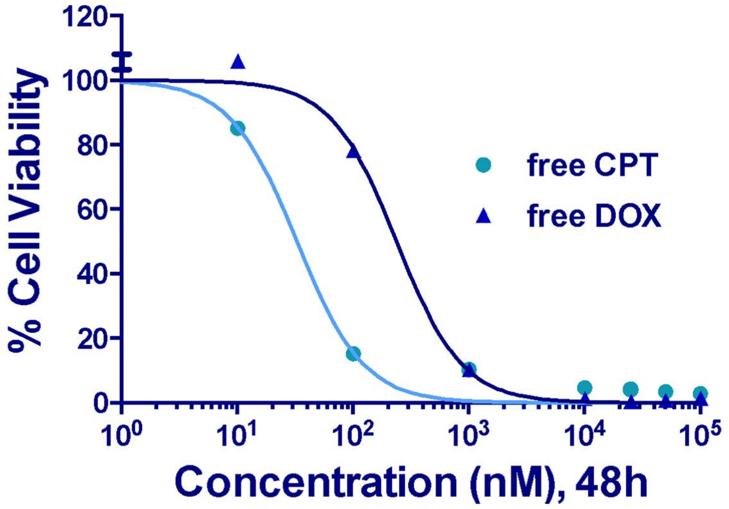

Figure S2. Dose-response curves and IC50 values for free DOX and free CPT treatment with HL-60.

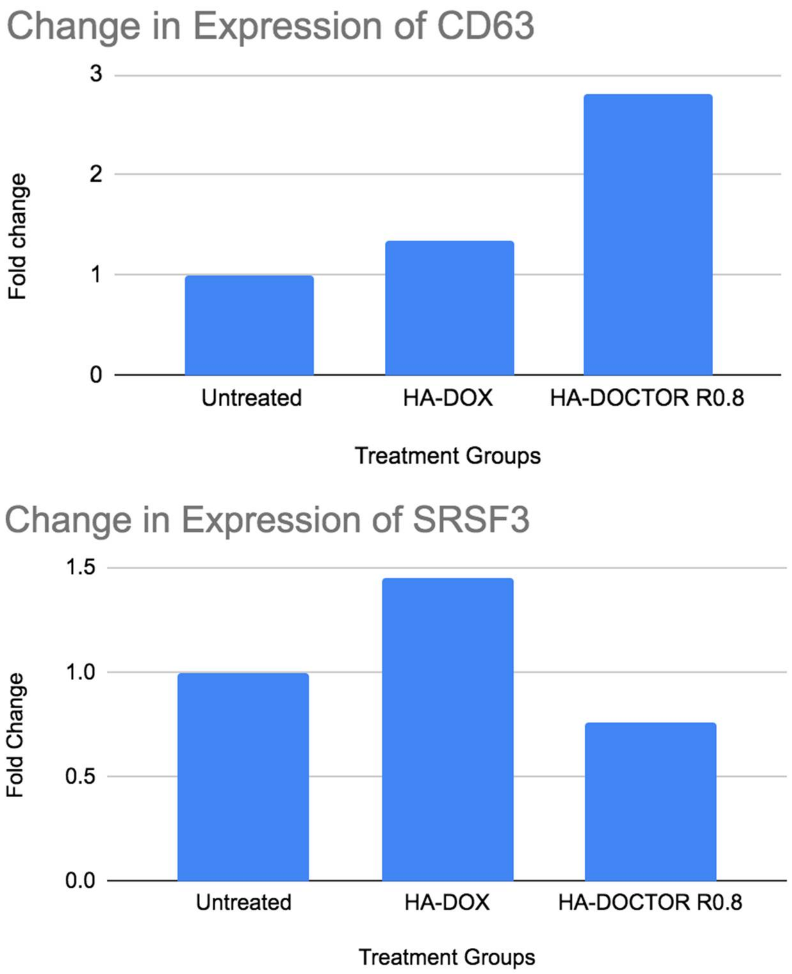

Figure S3. QPCR Validation. The mRNA expression of two representative proteins (SRSF3 and CD63) from the proteomics list was validated.

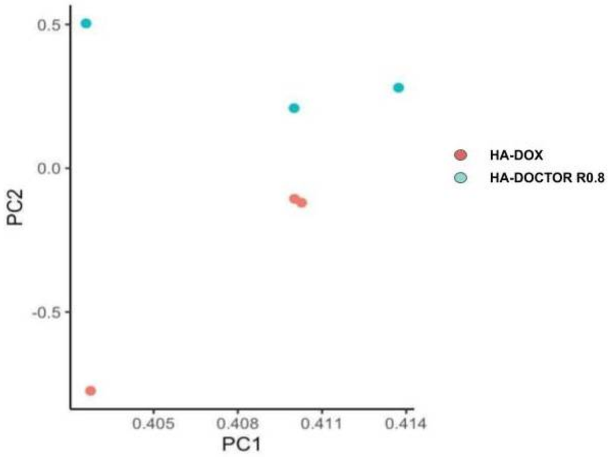

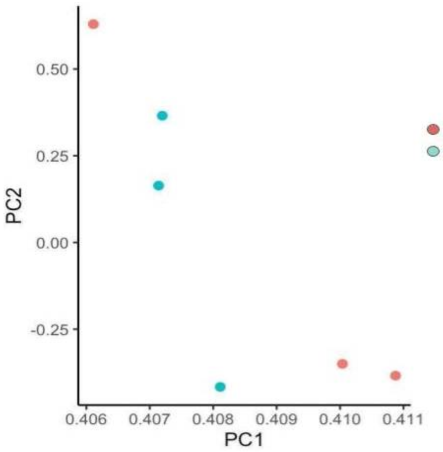

Figure S4.Principal Component Analysis was performed to bring out strong patterns within the dataset.

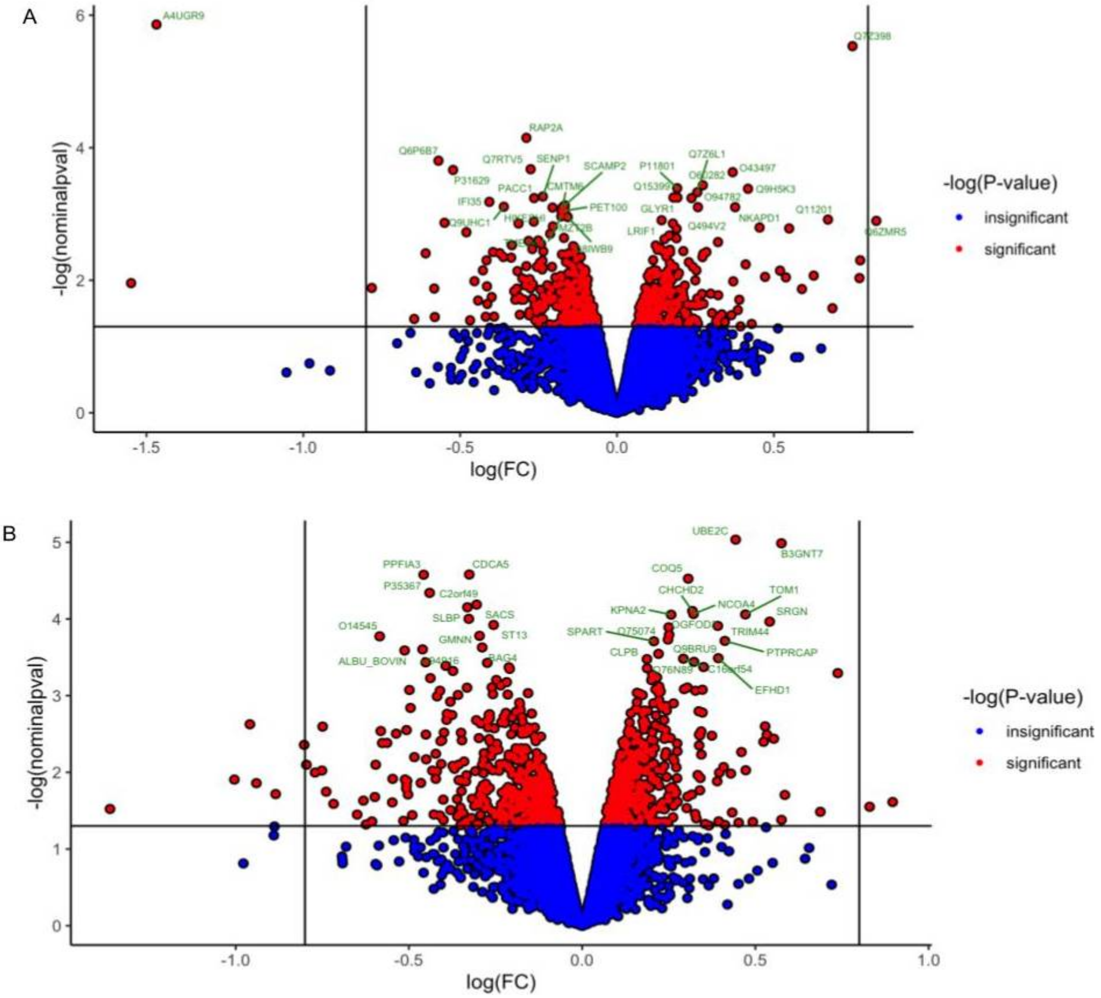

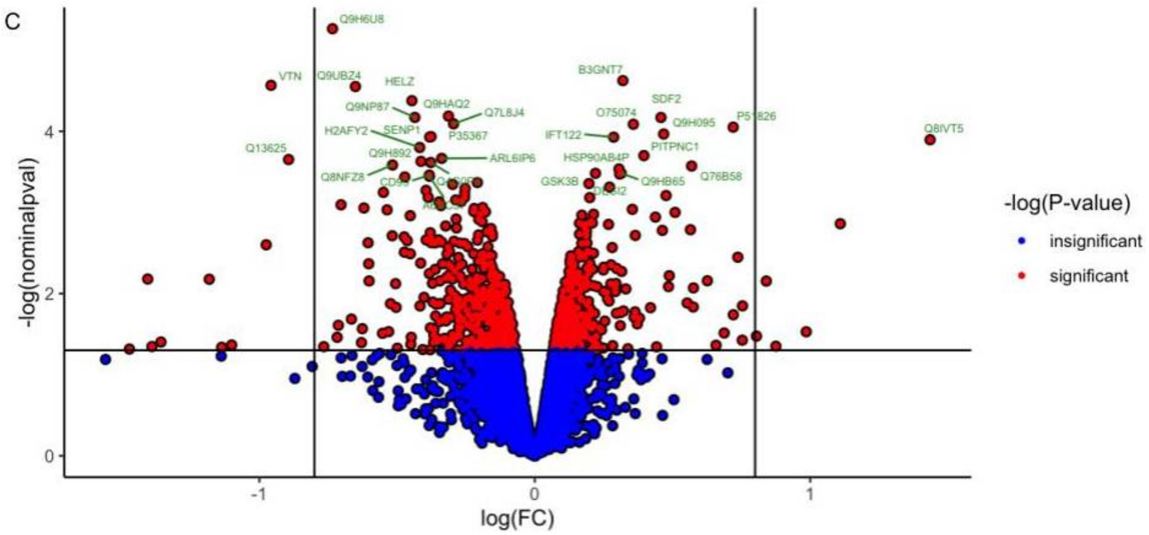

Figure S5. Differentially expressed proteins in treated vs untreated samples. Volcano plot illustrates significantly differentially abundant proteins. The −log10 (Benjamini–Hochberg corrected *P* value) is plotted against the log2 (fold change). (**A**) Untreated vs HA-DOX (**B**) Untreated vs HA-DOX-CPT R0.8 (**C**) Untreated vs HA-DOX-CPT R1.5.

Table S1. The drugs were incorporated onto HA in a series of molar ratios by varying the amount of drugs added and reaction time.

DOX:CPT (Ratio, R)	HA (mmoles)	DOX (mmoles)	CPT (mmoles)
**0.8:1**	0.05	0.006	0.021
**1.5:1**	0.05	0.01	0.021
**12:1**	0.05	0.05	0.006
**21:1**	0.05	0.05	0.003

Table S2. List of cell lines used.

Cell name	Description
HL-60	Acute myeloid leukemia
HL-60/MX2	Multidrug-resistant Acute myeloid leukemia
MV4;11	Acute monocytic leukemia
MOLM-13	Acute myeloid leukemia
MOLM-14	Acute myeloid leukemia
THP-1	Acute monocytic leukemia
K562	BCR-ABL1 positive chronic myelogenous leukemia
SUP-M2	Alk positive - anaplastic large cell lymphoma
DL-40	Alk negative - anaplastic large cell lymphoma
L82	Alk positive - anaplastic large cell lymphoma
KiJK	Alk positive - anaplastic large cell lymphoma
FEPD	Alk negative - anaplastic large cell lymphoma
HUT-78: CTCL	Human T cell lymphoma
MTA: NK-LL	NK/T-cell lymphoma

Table S3. Dose-response curves and IC50 values for free DOX and free CPT treatment with HL-60.

Table 3	Free DOX (nM)	Free CPT (nM)
IC90/HL-60 (48 h)	993 ± 0.16	141 ± 0.15

Table S4. Summary of IC90 and C.I. values for HA-DOX-CPT when treated with Acute Myeloid Leukemia cells (HL-60). HA-DOX-CPT achieves a high synergy with C.I values <1.

HA-DOX-CPT HL-60, 72 h	IC90	~CPT Fold Reduction	~DOX Fold Reduction	~C.I.
0.8	96 nM	4×	>10×	0.3(Synergistic)
1.5	401 nM	2×	>10×	0.6(Synergistic)
12.0	1 µM	6×	>10×	0.2(Synergistic)
21.0	2 µM	5×	>10×	0.2(Synergistic)

Table S5. Summary of IC90 and C.I. values for HA-DOX-CPT when treated with drug-resistant Acute Myeloid Leukemia cells (HL-60/MX2). DOCTOR achieves a very high synergy with C.I values <<1.

HA-DOX-CPT HL-60/MX2, 72 h	IC90	~CPT Fold Reduction	~DOX Fold Reduction	~C.I.
0.8	7 nM	12×	>10×	0.09 (Synergistic)
1.5	65 nM	2×	>10×	0.4(Synergistic)
12.0	635 nM	2×	>10×	0.5(Synergistic)
21.0	653 nM	3×	>10×	0.3(Synergistic)

Table S6. Summary of IC50 and C.I. values for DOCTOR when treated with Acute Monocytic Leukemia Cells (MV4; 11). HA-DOX-CPT achieves high synergy with C.I values <1.

HA-DOX-CPT MV4;11, 72 h	IC90	~CPT Fold Reduction	~DOX Fold Reduction	~C.I.
0.8	137 nM	6×	>10×	0.3 (Synergistic)
1.5	377 nM	4×	>10×	0.6(Synergistic)
12.0	3 µM	4×	>10×	0.2(Synergistic)
21.0	3 µM	8×	>10×	0.2(Synergistic)

Table S7. Summary of IC90 and C.I. values for DOCTOR when treated with Acute Myeloid Leukemia Cells (MOLM-13). HA-DOX-CPT achieves high synergy for ratios R0.8 and R1.5 with C.I values <1.

HA-DOX-CPT MOLM-13, 72 h	IC90	~CPT Fold Reduction	~DOX Fold Reduction	~C.I.
0.8	13 nM	2×	>10×	0.5 (Synergistic)
1.5	27 nM	2×	>10×	0.5(Synergistic)
12.0	621 nM	1×	>10×	1.5(Antagonistic)
21.0	985 nM	1×	>10×	1.4(Antagonistic)

Table S8. Summary of IC90 and C.I. values for DOCTOR when treated with Acute Myeloid Leukemia Cells (MOLM-14). HA-DOX-CPT achieves high synergy for ratios R0.8 and R1.5 with C.I values <1.

HA-DOX-CPT MOLM-14, 72 h	IC90	~CPT Fold Reduction	~DOX Fold Reduction	~C.I.
0.8	6 nM	2×	>10×	0.7 (Synergistic)
1.5	9 nM	2×	>10×	0.5(Synergistic)
12.0	253 nM	1×	>10×	2(Antagonistic)

21.0	345 nM	1×	>10×	1.5(Antagonistic)

Table S9. Summary of IC90 and C.I. values for DOCTOR when treated with Acute Monocytic Leukemia Cells (THP-1). HA-DOX-CPT achieves high synergy for ratios R0.8 and R1.5 with C.I values <1.

HA-DOX-CPT THP-1, 72 h	IC90	˜CPT Fold Reduction	˜DOX Fold Reduction	˜C.I.
0.8	6 nM	2×	>10×	0.5 (Synergistic)
1.5	9 nM	1.4×	>10×	0.7(Synergistic)
12.0	253 nM	1×	>10×	1(Additive)
21.0	345 nM	0.6×	>10×	2(Antagonistic)

Table S10. Summary of IC90 and C.I. values for DOCTOR when treated with BCR-ABL1+ Chronic Myeloid Leukemia Cells (K562). HA-DOX-CPT achieves very high synergy with C.I values <<1.

HA-DOX-CPT K562, 72 h	IC90	˜CPT Fold Reduction	˜DOX Fold Reduction	˜C.I.
0.8	89 µM	1.6×	>10×	0.6 (Synergistic)
1.5	44 µM	6×	>10×	0.2(Synergistic)
12.0	47 µM	45×	>10×	0.02(Synergistic)
21.0	49 µM	76×	>10×	0.01(Synergistic)

Table S11. Summary of IC90 and C.I. values for DOCTOR when treated with Alk+ anaplastic large cell lymphoma (SUP-M2). HA-DOX-CPT achieves high synergy with C.I values <1.

HA-DOX-CPT SUP-M2, 48 h	IC90	˜CPT Fold Reduction	˜DOX Fold Reduction	˜C.I.
0.8	39 nM	3×	>10×	0.4 (Synergistic)
1.5	41 nM	5×	>10×	0.2(Synergistic)
12.0	240 nM	7×	>10×	0.2(Synergistic)
21.0	467 nM	6×	>10×	0.2(Synergistic)

Table S12. Summary of IC90 and C.I. values for DOCTOR when treated with Alk- anaplastic large cell lymphoma (DL-40). HA-DOX-CPT achieves high synergy with C.I values <1.

HA-DOX-CPT DL-40, 48 h	IC90	˜CPT Fold Reduction	˜DOX Fold Reduction	˜C.I.
0.8	7 nM	12×	>10×	0.4 (Synergistic)
1.5	9 nM	17×	>10×	0.2(Synergistic)
12.0	97 nM	13×	>10×	0.2(Synergistic)
21.0	835 nM	11×	>10×	0.2(Synergistic)

Table S13. Summary of IC90 and C.I. values for DOCTOR when treated with Alk+ anaplastic large cell lymphoma (L82). HA-DOX-CPT achieves high synergy for ratios R0.8 and R1.5 with C.I values <1.

HA-DOX-CPT L82, 48 h	IC90	˜CPT Fold Reduction	˜DOX Fold Reduction	˜C.I.
0.8	28 nM	6×	>10×	0.2 (Synergistic)
1.5	73 nM	5×	>10×	0.2(Synergistic)
12.0	4 µM	0.7×	>10×	1.5(Antagonistic)
21.0	6 µM	0.8×	>10×	1.3(Antagonistic)

Table S14. Summary of IC90 and C.I. values for DOCTOR when treated with Alk+ anaplastic large cell lymphoma (Ki-JK). HA-DOX-CPT achieves high synergy for ratios R0.8, R1.5 and R12 with C.I values <1.

HA-DOX-CPT KiJK, 48 h	IC90	˜CPT Fold Reduction	˜DOX Fold Reduction	˜C.I.
0.8	21 nM	3×	>10×	0.4 (Synergistic)
1.5	46 nM	2×	>10×	0.5(Synergistic)
12.0	506 nM	2×	>10×	0.6(Synergistic)
21.0	2 µM	1×	>10×	1.4(Antagonistic)

Table S15. Summary of IC90 and C.I. values for DOCTOR when treated with Alk- anaplastic large cell lymphoma (FEPD). HA-DOX-CPT achieves high synergy for ratios R1.5, R12 and R21 with C.I values <1.

HA-DOX-CPT FEPD, 48 h	IC90	˜CPT Fold Reduction	˜DOX Fold Reduction	˜C.I.
0.8	17 nM	1×	>10×	1.2 (Antagonistic)
1.5	9 nM	3×	>10×	0.3(Synergistic)
12.0	113 nM	2×	>10×	0.6(Synergistic)
21.0	214 nM	2×	>10×	0.6(Synergistic)

Table S16. Summary of IC90 and C.I. values for DOCTOR when treated with T-cell lymphoma (HUT-78:CTCL). HA-DOX-CPT achieves very high synergy for all ratios with C.I values <<1.

HA-DOX-CPT HUT-78:CTCL, 48h	IC90	˜CPT Fold Reduction	˜DOX Fold Reduction	˜C.I.
0.8	404 nM	2×	>10×	Highly synergistic
1.5	807 nM	2×	>10×	Highly synergistic
12.0	4 µM	3×	>10×	Highly synergistic
21.0	4 µM	5×	>10×	Highly synergistic

Table S17. Summary of IC90 and C.I. values for DOCTOR when treated with NK/T-cell lymphoma (MTA:NK-LL). HA-DOX-CPT achieves very high synergy for all ratios with C.I values <<1.

HA-DOX-CPT MTA:NK-LL, 48 h	IC90	˜CPT Fold Reduction	˜DOX Fold Reduction	˜C.I.
0.8	6 nM	0.02×	>10×	>>>! (Highly Antagonistic)
1.5	8 nM	0.03×	>10×	>>>! (Highly Antagonistic)
12.0	85 nM	0.02×	>10×	>>>! (Highly Antagonistic)
21.0	158 nM	0.02×	>10×	>>>! (Highly Antagonistic)

Table S18. Select protein expression level comparison between HA-DOX and HA-DOX-CPT treated HL-60/MX2 identified by MS. Proteins upregulated in HA-DOX treatment, downregulated in HA-DOX-CPT R0.8 treatment (Orange). Proteins downregulated in HA-DOX treatment, upregulated in HA-DOX-CPT R0.8 treatment (Blue).

Protein symbol	Accession no	logFC	P.Value	adj.P.Val	FC
UBE2C	O00762	0.4460381404	0.0005	0.1135079049	1.178780071
HCK	P08631	0.4460381404	0.0005	0.1135079049	1.178780071
PARP1	P09874	−0.4479515105	0.0004	0.1014295581	−1.180831232
CDC5L	Q99459	−0.2917023084	0.0004	0.1014295581	−1.019849398

DDX21	Q9NR30	−0.4320615448	0.0002	0.1000606193	−1.163859617
PRPF3	O43395	−0.3516134476	0.0002	0.1000606193	−1.080060052
MTA2	O94776	−0.2546312959	0.0003	0.1000606193	−0.9834804749
AHCTF1	Q8WYP5	−0.2821078326	0.0004	0.1014295581	−1.010373017
RREB1	Q92766	−0.5336812874	0.0002	0.09620541093	−1.274920884
RFC1	P35251	−0.4535969867	0.0002	0.09620541093	−1.186895383
DNMT1	P26358	−0.3576600781	0.0002	0.09620541093	−1.086238648
UHRF1	Q96T88	−0.3205797224	0.0002	0.1000606193	−1.048645259

Table S19. Select protein expression level comparison between HA-DOX-CPT R0.8 and HA-DOX-CPT R1.5 treated HL-60/MX2 identified by MS. Proteins upregulated in HA-DOX-CPT R0.8 treatment, downregulated in HA-DOX-CPT R1.5 treatment (Yellow). Proteins downregulated in HA-DOX-CPT R0.8 treatment, upregulated in HA-DOX-CPT R1.5 treatment (Green).

Protein symbol	Accession no	logFC	P.Value	adj.P.Val	FC
SACS	Q9NZJ4	0.3346999833	0.0005	0.3497103337	1.062877871
METTL21A	Q8WXB1	0.5720247552	0.003	0.5495071319	1.318440477
SRGN	P10124	−0.5866405117	0.00003	0.1877787186	−1.33527275
UBE2C	O00762	−0.4240619723	0.0001	0.3405551606	−1.155369063
CHCHD2	Q9Y6H1	−0.2947821597	0.0003	0.3405551606	−1.022900884
HECTD1	Q9ULT8	−0.2657369911	0.0004	0.3497103337	−0.9943066241
TRIM44	Q96DX7	−0.3000591407	0.0007	0.3603241685	−1.028140108
APLP2	Q06481	−0.2596946688	0.0007	0.3603241685	−0.9884091287
B3GNT7	Q8NFL0	−0.2534026639	0.001	0.4846378327	−0.9822863613
NCOA4	Q13772	−0.2749716536	0.001	0.4846378327	−1.003353676
KAT7	O95251	−0.1725002038	0.002	0.5426893837	−0.9051840012
CD4	P01730	−0.03353779162	0.5	0.9356636827	−0.7792635606
KPNA2	P52292	−0.227254589	0.001	0.4846378327	−0.9570404525
C16orf54	Q6UWD8	−0.2525126954	0.002	0.5009142589	−0.9814218415
ALCAM	Q13740	−0.1861308509	0.003	0.5495071319	−0.917968453
MYB	P10242	−0.2869998708	0.003	0.5495071319	−1.015199231

Table S20. Primer sequences for qPCR validation.

Gene	Primer Sequence
Beta Actin	F: GCAAAGACCTGTACGCCA
	R: TGCATCCTGTCGGCAATG
CD63	F: TAGATTCGGCAGCCATGGCGGTGGAA
	R: ACTGACCAGACCCCTACATCACC
SRSF3	F: GGCAATCTTGGAAACAATGG
	R : TTCACCATTCGACAGTTCCA
